# Sporadic Gene Loss After Duplication Is Associated with Functional Divergence of Sirtuin Deacetylases Among *Candida* Yeast Species

**DOI:** 10.1534/g3.116.033845

**Published:** 2016-08-18

**Authors:** Christopher B. Rupert, Justin M. H. Heltzel, Derek J. Taylor, Laura N. Rusche

**Affiliations:** Department of Biological Sciences, State University of New York at Buffalo, New York 14260

**Keywords:** Sir2, Hst1, specifically retained ancestral gene, CTG clade

## Abstract

Gene duplication promotes the diversification of protein functions in several ways. Ancestral functions can be partitioned between the paralogs, or a new function can arise in one paralog. These processes are generally viewed as unidirectional. However, paralogous proteins often retain related functions and can substitute for one another. Moreover, in the event of gene loss, the remaining paralog might regain ancestral functions that had been shed. To explore this possibility, we focused on the sirtuin deacetylase *SIR2* and its homolog *HST1* in the CTG clade of yeasts. *HST1* has been consistently retained throughout the clade, whereas *SIR2* is only present in a subset of species. These NAD^+^-dependent deacetylases generate condensed chromatin that represses transcription and stabilizes tandemly repeated sequences. By analyzing phylogenetic trees and gene order, we found that a single duplication of the *SIR2/HST1* gene occurred, likely prior to the emergence of the CTG clade. This ancient duplication was followed by at least two independent losses of *SIR2*. Functional characterization of Sir2 and Hst1 in three species revealed that these proteins have not maintained consistent functions since the duplication. In particular, the rDNA locus is deacetylated by Sir2 in *Candida albicans*, by Hst1 in *C. lusitaniae*, and by neither paralog in *C. parapsilosis*. In addition, the subtelomeres in *C. albicans* are deacetylated by Sir2 rather than by Hst1, which is orthologous to the sirtuin associated with *Saccharomyces cerevisiae* subtelomeres. These differences in function support the model that sirtuin deacetylases can regain ancestral functions to compensate for gene loss.

Gene duplication is a powerful force driving protein evolution. After duplication, two evolutionary paths lead to increased protein diversity. Neofunctionalization occurs when one duplicate develops a completely new function, whereas subfunctionalization occurs when the duplicated genes each retain a subset of the ancestral functions. These fates of duplicated genes have been modeled extensively (Conant and Wolfe 2008; Hahn 2009; Espinosa-Cantu *et al.* 2015), but less attention has been given to the consequences of gene loss that occurs after paralogs have diverged. To explore the biological impact of such loss, we focused on the sirtuin deacetylase *SIR2* and its homolog *HST1* (Homolog of Sir Two) in the CTG clade of budding yeast. In this case, *HST1* has been consistently retained throughout the clade, whereas *SIR2* has been sporadically lost.

Sirtuins are NAD^+^-dependent deacetylases found in all domains of life (Greiss and Gartner 2009). These deacetylases are of particular interest because they require the metabolite NAD^+^ for activity, and are therefore proposed to be sensitive to the metabolic state of a cell and hence nutrient availability. In budding yeasts, Sir2 and its homologs affect chromatin structure by deacetylating histone tails. Deacetylation changes the charge of a lysine from neutral to positive, thereby increasing electrostatic interactions of the histone tails with negatively charged DNA and other nucleosomes. Thus, the action of Sir2 leads to a compact chromatin state and the repression of transcription. In *Saccharomyces cerevisiae*, Sir2 is the key enzyme that forms heterochromatin at the mating-type loci (Rine and Herskowitz 1987) and subtelomeres (Gottschling *et al.* 1990). Sir2 also stabilizes the copy number of the rDNA repeats (Gottlieb and Esposito 1989). *SIR2* has a paralog, *HST1*, which arose through a whole genome duplication in the *Saccharomyces* lineage. Following duplication, these genes subfunctionalized (Hickman and Rusche 2009; Froyd and Rusche 2011). In *S. cerevisiae*, Hst1 represses genes required for sporulation and NAD^+^ biosynthesis (Xie *et al.* 1999; Bedalov *et al.* 2003). Interestingly, despite having subfunctionalized, Sir2 and Hst1 in *S. cerevisiae* retain a modest ability to substitute for one another (Xie *et al.* 1999; Hickman and Rusche 2007).

Sir2 and Hst1 contribute to the pathogenicity of *Candida glabrata*, a yeast in the *Saccharomyces* clade. In *C. glabrata*, Sir2 and Hst1 both repress genes that promote virulence, including cell-adhesion and stress-resistance genes (Gallegos-Garcia *et al.* 2012; Orta-Zavalza *et al.* 2013). Moreover, because *C. glabrata* is unable to synthesize NAD^+^ *de novo* (Li and Bao 2007), the activities of Sir2 and Hst1 are reduced in environments low in NAD^+^ precursors, causing virulence-promoting genes to be induced (Domergue *et al.* 2005). Unlike *C. glabrata*, most pathogenic yeast belong to the CTG clade, which is named for a switch of the CTG codon from leucine to serine (Santos *et al.* 1993). This clade contains multiple human pathogens that account for approximately three-fourths of nosocomial fungal infections (Azie *et al.* 2012). The species responsible for most infections is *C. albicans*, but species such as *C. parapsilosis* are increasing in prevalence (Yapar 2014). The contributions of Sir2 and Hst1 to the virulence of these species are not well-characterized.

A duplication of *SIR2/HST1* is evident in the CTG clade and is distinct from the whole-genome duplication that gave rise to *SIR2* and *HST1* in *S. cerevisiae* and *C. glabrata*. This CTG duplication raises several conundrums. First, not all CTG species encode both paralogs, and those species that have both copies do not fall in a monophyletic group. Consequently, it is unclear how many duplications and losses of *SIR2/HST1* occurred in the CTG lineage. Another puzzle is that in *C. lusitaniae*, which lacks Sir2, the remaining paralog Hst1 has a surprisingly restricted role compared to orthologous proteins in other yeast (Froyd *et al.* 2013; Kapoor *et al.* 2015). ClHst1 acts at the rDNA, but not at subtelomeres, sporulation genes, or centromeres. This restricted role for ClHst1 suggests a model in which the loss of *SIR2* occurred after subfunctionalization had taken place, leaving *C. lusitaniae* with a reduced set of sirtuin-mediated functions.

To clarify the evolutionary history of the *SIR2*/*HST1* gene family in the CTG clade, we determined how many duplications and losses occurred. By analyzing phylogenetic trees and gene order for a larger set of species, we found that a single duplication was followed by at least two independent losses of the *SIR2* ortholog. We also investigated the functional consequences of gene loss by comparing the genomic loci at which Sir2 and Hst1 act in three species. Surprisingly, Sir2 and Hst1 have not maintained consistent functions since duplication. Thus, gene loss during protein diversification adds complexity to the fates of genes originating from the same duplication.

## Materials and Methods

### Gene sequences, alignment, and tree construction

Amino acid sequences of Sir2 and Hst1 (Supplemental Material, Table S1) were obtained from the *Candida* gene order browser (Maguire *et al.* 2013), the Yeast Gene order browser (Byrne and Wolfe 2005), GenBank, and by tBLASTn searches of genomic assemblies of *Scheffersomyces coipomoensis* (Taylor *et al.* 2013) and *S. spartinae* (strain NRRL Y-7322). *S. spartinae* was sequenced from a DNA library using 454 Life Sciences (http://www.454.com) methods and GS FLX Titanium series reagents. We obtained 314,299 reads with approximately 133 Mb of aligned bases. Newbler (http://www.454.com) was used for assembly, resulting in 11.8 Mb (1511 contigs) with an average peak depth of 7 ×. Gene sequences from *S. coipomoensis* and *S. spartinae* were deposited in GenBank (accession numbers KX533520–KX533543).

For the protein tree, sequences were aligned using the MAFFT E-INS-i algorithm with default settings (Katoh and Standley 2013). The best fit substitution model was determined to be WAG+I+F+G using the Bayesian information criterion (BIC) with Partionfinder Protein (Lanfear *et al.* 2012). The fully aligned Sir2 and Hst1 sequences were used to estimate a maximum likelihood (ML) tree using PhyML 3.1 as implemented in Seaview 4.6 (Gouy *et al.* 2010). Node support was assessed with approximate likelihood ratio tests (aLRT). Subtree pruning and regrafting (SPR) was used as a search algorithm with five random starts. Hst2 sequences from *C. albicans* and *S. cerevisiae* were used for outgroup rooting. All phylogenetic analyses used methods that are free of clock assumptions (Wertheim *et al.* 2010).

For the species tree, we used 10 single copy genes (Table S2) that have been identified as among the most phylogenetically informative for yeast species (Aguileta *et al.* 2008) to estimate the relationships of *Scheffersomyces* species to other CTG clade members. Alignment of amino acids was carried out in MAFFT using E-INS-i with default settings. Gblocks (Castresana 2000) was applied as a filter for unreliable sites. Partitionfinder Protein was used to assess substitution models with partitions designated for each gene. ML estimation of a concatenated gene tree was carried out in RAxML 8.1.2 (Stamatakis 2014). Removing all outgroup taxa and their potential biases yielded a similar tree to the outgroup-rooted tree. A Coalescent-based species tree with local posterior probabilities was estimated using ASTRAL 4.10.7 (Sayyari and Mirarab 2016).

To assess gene copy number changes from species phylogenies, we searched for the best ML tree with a single node constrained to reflect a scenario of one loss of Sir2 (in the *Lodderomyces* clade). An SH (Shimodaira–Hasegawa)-test was carried out in RAxML to determine if the score of the best “one gene loss” tree was significantly inferior to the score of the observed ML tree.

For synteny analysis, we used the *Candida* Gene Order Browser (Maguire *et al.* 2013), an online tool that allows visualization of gene synteny across multiple CTG clade species.

### Yeast growth and transformation

*C. albicans* and *C. parapsilosis* (Table 1) were derived from established laboratory strains (Noble and Johnson 2005; Holland *et al.* 2014) and grown in YPD (1% yeast extract, 2% peptone, and 2% dextrose) at 30°. Strains generated for this study are available upon request. To select for auxotrophic markers, transformed cells were recovered on synthetic dropout plates [0.67% yeast nitrogen base, 2% dextrose, and CSM supplements (CSM-LEU, BIO 101 4512-422; CSM-HIS, BIO 101 4512-222; or CSM–ARG Sunrise Science 1031-100)].

**Table 1:** Strains used in this study

Strain Name	Genotype	Source
SN152	*C. albicans his1*::*hisG/his1*::*hisG leu2/leu2 arg4/arg4 ura3*::*imm434*::*URA3/ura3*::*imm434 iro1*::*IRO1/iro1*::*imm434*	Noble and Johnson, 2005
LRY3045	SN152 *sir2*Δ::*ARG4/sir2*Δ::*LEU2*	This study
LRY3046	SN152 *sir2*Δ::*ARG4/sir2*Δ::*LEU2*	This study
LRY3047	SN152 *hst1*Δ::*ARG4/hst1*Δ::*LEU2*	This study
LRY3048	SN152 *hst1*Δ::*ARG4/hst1*Δ::*LEU2*	This study
CPL2H1	*C. parapsilosis leu2 his1*	Holland *et al.* 2014
LRY3083	CPL2H1 *hst1*Δ::*HIS1/hst1*Δ::*LEU2*	This study
LRY3084	CPL2H1 *hst1*Δ::*HIS1/hst1*Δ::*LEU2*	This study

Both *C. albicans* and *C. parapsilosis* were transformed by electroporation using a modification of (Froyd *et al.* 2013). Overnight cultures were diluted to OD_600_ = 0.4 in YPD and grown for 4–5 hr to OD_600_ = 3. 50 OD of cells were collected, resuspended in 10 ml 0.1 M LiOAc, 10 mM Tris pH 8, 1 mM EDTA, and 10mM DTT, and incubated with shaking for 1 hr at 30°. Cells were collected and resuspended in 500 μl 1 M sorbitol. A total of 40 μl cells were mixed with the transformation cassette (8 μg for *C. albicans* and 2 μg for *C. parapsilosis*) and electroporated at 1800 V, 25 µF, 200 Ω. Electroporated samples were immediately resuspended in 1 ml YPD and incubated with shaking at 30° for 4 hr. Cells were collected and plated on selective medium.

### Strain construction

To delete *HST1* and *SIR2*, we first generated knockout cassettes on plasmids (Table S3). The *SIR2* and *HST1* genes, including approximately 1000 bp upstream and downstream, were amplified by PCR and then ligated into pRS316 (Sikorski and Hieter 1989). Next, knockout cassettes were created by replacing the open reading frames with one of three selectable markers. *ARG4*, *LEU2*, or *HIS1* were amplified from pSN69, pSN40, or pSN52 (Noble and Johnson 2005) with bipartite primers that also included approximately 20 bp of sequence that flanks the open reading frames of *SIR2* or *HST1*. These PCR products were then used in a PCR stitching reaction (18 cycles of 98° for 40 sec, 62° for 50 sec, and 72° for 20 min) to replace the open reading frames. The products of PCR stitching reactions were digested with *Dpn*I and recovered through *Escherichia coli*.

Knockout cassettes for transformation in *C. albicans* were amplified from plasmids pLR996 (*sir2*Δ::*ARG4*) or pLR998 (*hst1*Δ::*ARG4*), or from strains DHCA254 (*sir2*Δ::*LEU2*) or DHCA250 (*hst1*Δ::*LEU2*) (Hnisz *et al.* 2009). Knockout cassettes for *C. parapsilosis* were from plasmids pLR1136 (*hst1*Δ::*HIS1*) and pLR1137 (*hst1*Δ::*LEU2*). Two independent deletions of the first allele were obtained, and then the second allele was deleted independently in each of these isolates. Deletions were confirmed by PCR.

### Chromatin immunoprecipitation (ChIP)

ChIP was essentially conducted as previously described (Froyd *et al.* 2013). Cells were diluted to OD_600_ = 0.4 and grown to a final OD of 3.0. 100 OD equivalents of cells were cross-linked in 1% formaldehyde, collected, and stored at –80°. Cells were suspended in 200 µl lysis buffer with glass beads (Biospec 11079105z) and lysed by agitation in a FastPrep bead beater (Model FP120A-115) for four 30 sec pulses. The lysate was transferred to fresh tubes in a final volume of 800 µl lysis buffer. Chromatin was sheared by sonication three times for 10 sec using an MSE soniprep 150. Immunoprecipitation (IP) was performed using 3–5 µl antibody [Anti-Histone H3 CT clone A3S (Millipore 05-928) or Anti-acetyl-Histone H3 (Lys9) (Millipore 06-942)] and incubated with rotation for 1 hr (*C. albicans*) or overnight (*C. parapsilosis*) at 4°. Antibody-protein complexes were captured for 1 hr at 4° with 60 µl Protein A agarose beads (Millipore 16-125) blocked with 0.1 mg/ml salmon sperm DNA and 0.25 mg/ml BSA.

To determine change in acetylation, we first measured the recovery of each amplicon (Table S4) using real time PCR. DNA in the IP samples was quantified relative to a standard curve prepared from input DNA, and this value was normalized to a control locus (*PRI2* in *C. parapsilosis* and an intergenic region between orf19.2926 and orf19.2927 in *C. albicans*). The relative recovery of each amplicon was determined for at least two independent IPs from two independently constructed strains, with each IP analyzed twice by real-time PCR. The average relative recovery was calculated separately for the total H3 and acetyl-H3K9 IPs for each strain. Next, for each amplicon, we calculated the ratio of recovery in the acetyl-H3-K9 samples compared to the total histone H3 samples. Finally, the ratio of acetyl-H3K9 to total H3 for the *hst1*Δ*/*Δ or *sir2*Δ*/*Δ strains was normalized to the ratio for the wild-type strain. The SD of the ratios was calculated as Sr = N/D * sqrt[(Sn/N)^2^ + (Sd/D)^2^], where N = average of numerator measurements, Sn = SD of numerator measurements, D = average of denominator measurements, Sd = SD of denominator measurements, and Sr = SD of the ratio N/D.

### Data availability

Accession numbers of sequences are deposited in GenBank: KX533520–KX533543. 

## Results

### HST1 and SIR2 genes are separated by a single branch point

**Figure 1:**
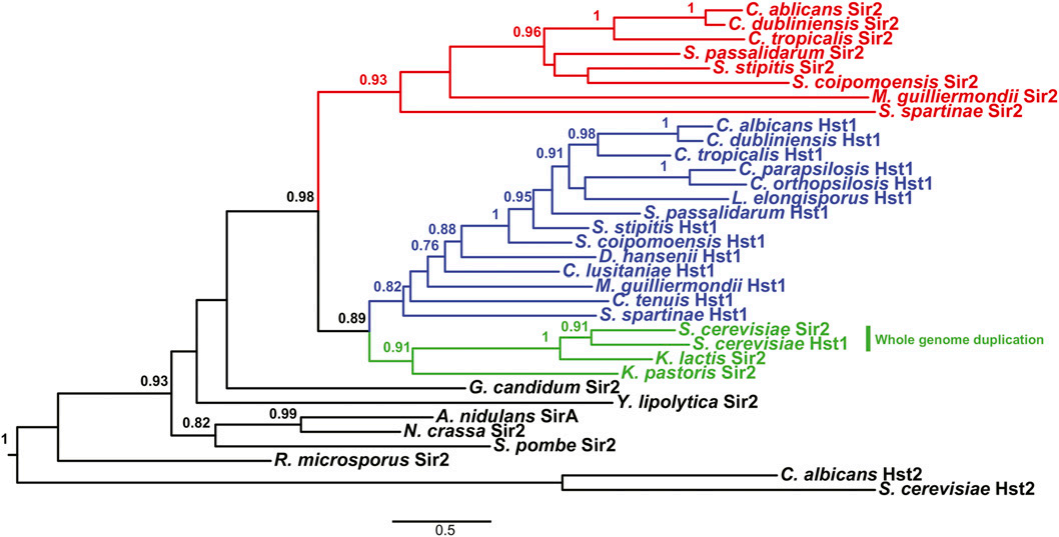
Sir2 and Hst1 are separated at a single branch point. An alignment of Hst1 and Sir2 amino acid sequences (Table S1) was used to create a ML (maximum likelihood) tree. Node support values (aLRT, approximate likelihood ratio test) above 0.75 are shown. Red indicates the CTG clade Sir2 sequences, blue indicates the CTG Hst1 sequences, and green represents Sir2 homologs from the *Saccharomyces* clade.

As a first approach to determine how many duplications of the *HST1/SIR2* gene occurred in the CTG lineage, we created a ML phylogenetic tree of Hst1 and Sir2 proteins. If a single duplication occurred, all Hst1 proteins would be separated from all Sir2 proteins at a single branch point. In contrast, if multiple duplications occurred, Hst1 and Sir2 proteins would be intermingled. The tree (Figure 1) revealed a single branch separating all CTG Sir2 proteins from CTG Hst1 proteins with strong node support (aLRT = 0.98). The separation of sequences at one branch point supports a single duplication leading to the paralogs *HST1* and *SIR2* in CTG species.

To estimate when the duplication occurred, the tree included Hst1/Sir2 proteins from yeast species outside the CTG clade, as well as more distant species of fungi. The CTG and *Saccharomyces* clades both belong to the subphylum Saccharomycotina. Sir2 proteins from non-Saccharomycotina species, such as *Schizosaccharomyces pombe*, *Neurospora crassa*, and *Aspergillus nidulans*, group outside the CTG Hst1 and Sir2 proteins. In addition, species which are basal in the Saccharomycotina, including *Yarrowia lipolytica* and *Geotrichum candidum*, grouped outside the CTG Hst1 and Sir2 proteins. Thus, the duplication occurred within the Saccharomycotina subphylum. In contrast, Sir2 proteins from species more closely related to the CTG clade, such as *S. cerevisiae*, *Kluyveromyces lactis*, and *Komagataella pastoris* clustered with the CTG Hst1 proteins and separately from the CTG Sir2 proteins (Figure 1). This result is consistent with the duplication occurring in a common ancestor of the CTG and *Saccharomyces* clades. However, it is also possible that the duplication occurred early in the CTG lineage and was followed by more rapid evolution of *SIR2* compared to *HST1*.

In contrast to the ancient duplication of the *HST1* and *SIR2* genes found in the CTG clade, the *HST1* and *SIR2* genes in *S. cerevisiae* arose more recently in a whole-genome duplication (Wolfe and Shields 1997). Indeed, ScHst1 and ScSir2 cluster together within the *Saccharomyces* clade (Figure 1). Thus, despite their identical names, CTG Sir2 is not orthologous to ScSir2. Instead, CTG Hst1 may be orthologous to both ScSir2 and ScHst1.

### HST1 is at the ancestral locus of the SIR2/HST1 gene

**Figure 2:**
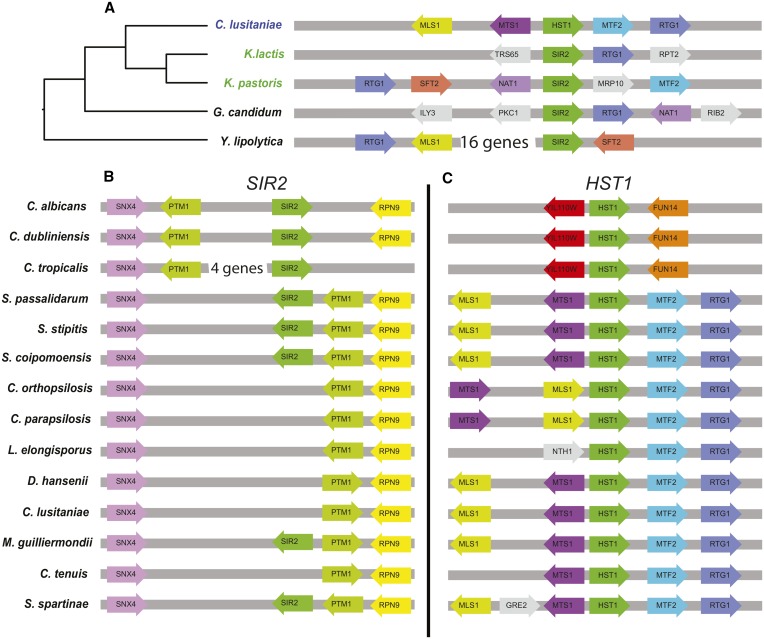
*HST1* is at the original locus of the *SIR2/HST1* gene. Gene order was determined using the *Candida* gene order browser (Maguire *et al.* 2013) and the comparative genomics CoGe browser (Lyons and Freeling 2008). Orthologous genes are indicated by a consistent color, and the orientation of the coding strand is indicated by the arrow. (A) Gene order surrounding *HST1/SIR2* is shown for representative species across the Saccharomycotina. (B) Gene order surrounding *SIR2* is shown for all CTG species examined. (C) Gene order surrounding *HST1* is shown for all CTG species examined.

Another line of evidence supporting a single duplication would be the presence of *HST1* and *SIR2* each at a consistent genomic locus across the CTG clade. If a gene duplicates once, the copy of the gene could remain at its new genomic locus in all descended species. In contrast, if a gene duplicates twice, the two independent copies are unlikely to be inserted at the same genomic location. Importantly, the genomic site of integration will remain evident even after species diverge because chromosomal rearrangements that alter gene order occur relatively infrequently (Byrne and Wolfe 2005; Maguire *et al.* 2013). To interpret the genomic positions of *HST1* and *SIR2*, we first determined which gene, *HST1* or *SIR2*, is at the ancestral locus. For this analysis, we searched for common genes surrounding the *HST1* and *SIR2* loci in CTG species compared with the nonduplicated *HST1/SIR2* locus in *Y. lipolytica* and *G. candidum*. We found that *HST1* is located near (within 18 genes) *RTG1* in the CTG species *C. lusitaniae*, as well as in *Y. lipolytica* and *G. candidum*. *HST1* is also near *MLS1* in *C. lusitaniae* and *Y. lipolytica* (Figure 2A). These same genes, *RTG1* and *MLS1*, are also close to *HST1* in other CTG species (Figure 2C). Finally, other genes, such as *SFT2* and *NAT1*, are near (within two genes) nonduplicated *SIR2/HST1* genes in the basal species *Y. lipolytica* and *G. candidum*, and in species with *SIR2/HST1* genes that cluster with CTG *HST1* genes, such as *K. pastoris* and *K. lactis* (Figure 2A). In contrast, the CTG *SIR2* gene does not share synteny with the *SIR2/HST1* genes in *Y. lipolytica* or *G. candidum*. Thus, the CTG *HST1* genes are in the ancestral locus of the *HST1/SIR2* gene, and therefore *SIR2* is the derived gene.

### The derived gene, SIR2, is at a consistent locus across the CTG clade

Having established that *HST1* is the ancestral copy of the *HST1/SIR2* gene, it is possible to use the genomic location of the derived gene, *SIR2*, to evaluate the number of duplications that occurred. If all the derived *SIR2* genes arose through a single duplication, gene synteny surrounding *SIR2* will likely be shared across CTG clade species. In contrast, if multiple duplications occurred, *SIR2* would be inserted into different genomic locations. In nearly all species, *SIR2* is adjacent to *PTM1* and within two genes of *SNX4* and *RPN9* (Figure 2B). The single exception is in *C. tropicalis*, in which an insertion of several genes separates *SIR2* from *PTM1*. Thus, *SIR2* most likely arose through a single duplication.

The genes surrounding *HST1* also show a high degree of shared synteny within the CTG clade (Figure 2C). In most species, *HST1* is adjacent to *MTF2* and *RTG1* on one side and *MTS1* and *MLS1* on the other side. However, in *C. dubliniensis*, *C. tropicalis*, *and C. albicans*, *HST1* is associated with different genes. Thus, it is likely that *HST1* moved to a new location in a common ancestor of these species.

### A species tree is consistent with at least two independent losses of SIR2 in CTG yeast

**Figure 3:**
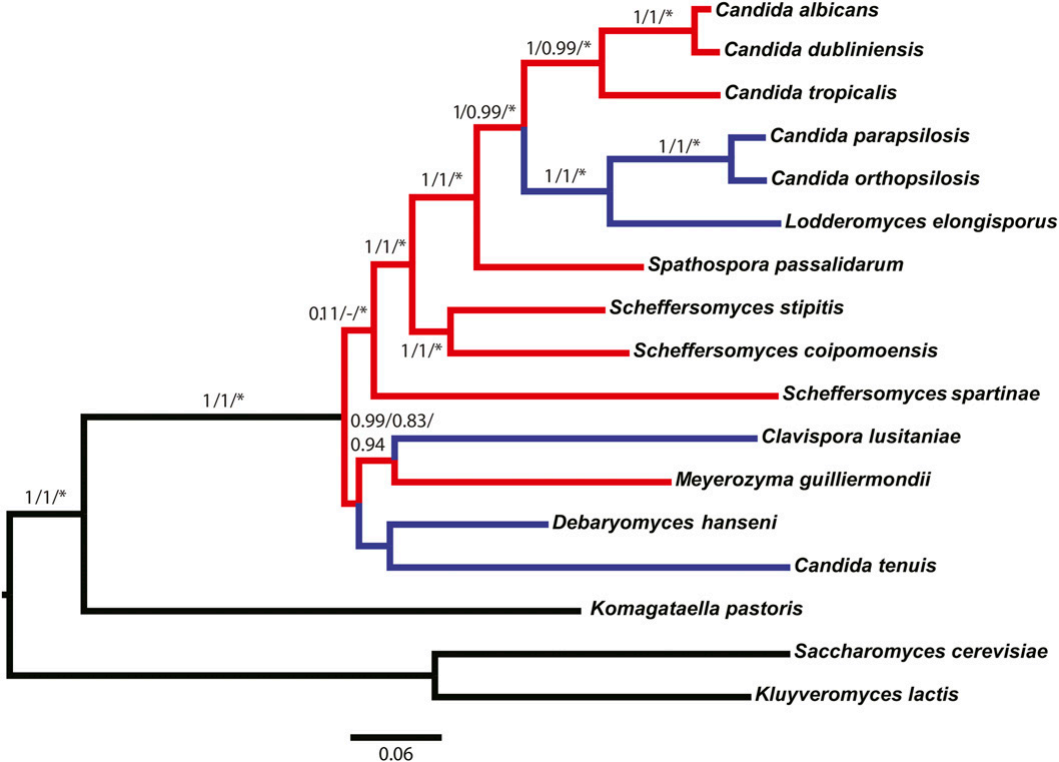
Tree showing the relationships of the CTG clade of fungi. Species containing *SIR2* are interspersed with species lacking *SIR2*. Branch support values are aLRTs (approximate likelihood ratio tests) for a concatenation tree/local posterior probabilities for a coalescent-based tree/strong nodes from gene trees (* indicates at least one gene tree with aLRT > 0.95). Red indicates CTG species containing both *SIR2* and *HST1*, and blue indicates CTG species containing only *HST1*.

Our preliminary analysis of CTG species suggested that those species with both *SIR2* and *HST1* genes are phylogenetically interspersed with species encoding only *HST1*. Therefore, *SIR2* was likely lost in some lineages. To determine how many losses occurred, we constructed a tree of the same species for which we analyzed Sir2 and Hst1 (Figure 3). This tree includes *S. spartinae*, which has not been analyzed previously. We found strong support for well-established relationships of CTG yeast. However, the phylogenetic positions of the basal species *D. hansenii*, *C. tenuis*, *C. lusitaniae*, *M. guilliermondii*, and *S. spartinae* remained weakly supported with short internodes, as in other studies (Wohlbach *et al.* 2011; Salichos and Rokas 2013; Hittinger *et al.* 2015). In addition, there was no evidence for monophyly of *Scheffersomyces* species. Indeed, the monophyly of *Scheffersomyces* is controversial and recent studies have proposed that the component species should be assigned to several genera (Kurtzman and Robnett 2013; Suh *et al.* 2013).

Based on this tree and the evidence that CTG *SIR2* arose through a single duplication, *SIR2* must have been lost in the *Lodderomyces* clade (*L. elongisporus*, *C. parapsilosis*, and *C. orthopsilosis*). These species lack *SIR2* but are situated within a larger clade of species with *SIR2*. A second independent loss of *SIR2* is indicated by the position of *M. guilliermondii*, which encodes both *SIR2 and HST1*. In this tree, *M. guilliermondii* and *C. lusitaniae* share a strongly supported branch. A slightly different topology can be obtained by removing the most distant ingroup species, *C. tenuis*. The average support for this tree is increased, and *M. guilliermondii* is placed with a different non-*SIR2* species, *D. hansenii*. However, in either case, *M. guilliermondii* is positioned away from other species with *SIR2* and *HST1*, indicating a more ancient timing for the duplication and, therefore, that *SIR2* must have been lost in the *C. lusitaniae* and *D. hansenii* lineages.

We also excluded the one-loss hypothesis using an SH-test in which all *SIR2*-containing species, including *M. guilliermondii*, were constrained within a single clade. This constraint, which leaves all nodes free to vary save one, forces the topology that would occur if *SIR2* were only lost once. However, it yielded a significantly worse tree than the observed ingroup tree [best tree ML score: −81877.628706; constraint tree ML score: −81910.227721; D(LH): -32.599015; SD: 12.327716; and *P* < 0.01]. Although short internodes and long branches appeared to affect support values and topologies, each outcome supported a scenario with two or more losses of the *SIR2* gene in CTG yeast.

Finally, we note that the apparent losses of *SIR2* are unlikely to be artifacts of genome assembly. Not only do the losses occur in multiple taxa, but the genomic loci that should be associated with *SIR2* are indeed present in these species (Figure 2B).

### CaSir2, but not CaHst1 or CpHst1, contributes to deacetylation at rDNA repeats

The analyses described above indicate that a single duplication of *HST1/SIR2* occurred in a common ancestor of CTG species and that, subsequent to this duplication, at least two independent losses of *SIR2* occurred. Notably in each case it is *SIR2*, and not *HST1*, that is lost, implying that there is a selective pressure to retain *HST1*. We previously reported that in *C. lusitaniae*, Hst1 acts only at the rDNA (Froyd *et al.* 2013; Kapoor *et al.* 2015). Therefore, it is possible that Hst1 has been selectively retained because it performs a critical function at the rDNA. If so, Hst1, but not Sir2, should function at the rDNA locus in other CTG species. To test this hypothesis, we determined whether Hst1 functions at the rDNA in two additional species, *C. albicans* and *C. parapsilosis*. *C. albicans* encodes both Hst1 and Sir2, whereas *C. parapsilosis* only encodes Hst1.

**Figure 4:**
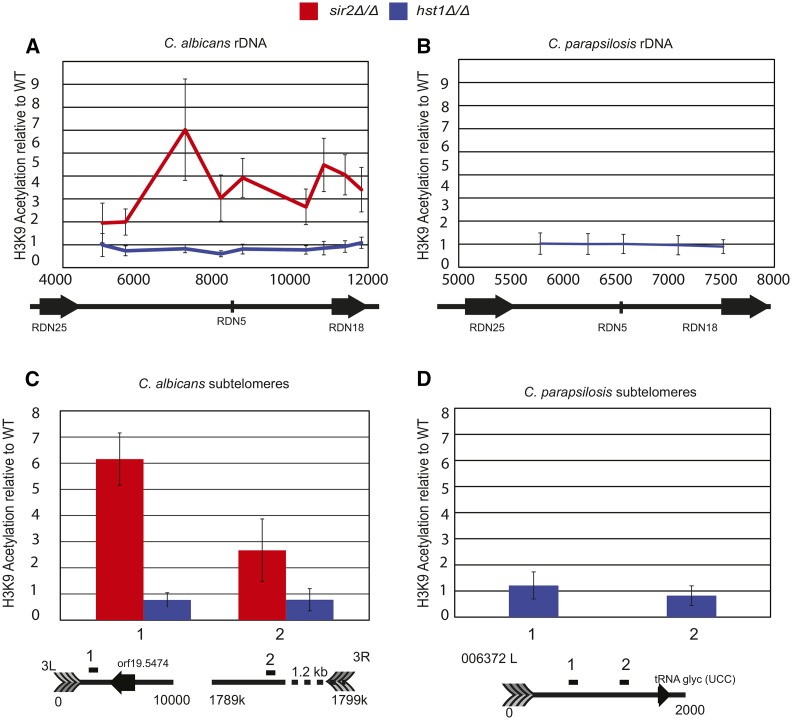
CaSir2, but not CaHst1 or CpHst1, contributes to deacetylation at rDNA repeats and subtelomeres. (A) The change in acetylation of H3K9 was determined across the rDNA nontranscribed spacer for *sir2*Δ*/*Δ (LRY3045 and LRY3046) or *hst1*Δ*/*Δ (LRY3047 and LRY3048) *C. albicans* strains compared to a wild-type strain (SN152). ChIP was performed using antibodies against acetyl-H3K9 or total H3, and the recovery of each rDNA amplicon was determined relative to a control locus. The recovery of acetyl-H3K9 was then normalized to total H3, and finally H3K9 acetylation in the deletion strains was normalized to a wild-type strain. (B) The change in acetylation of H3K9 was determined across the rDNA nontranscribed spacer for *hst1*Δ*/*Δ (LRY3083 and LRY3084) *C. parapsilosis* strains compared to a wild-type strain (CPL2H1), as described above. (C) The change in acetylation of H3K9 was determined at two subtelomeres using the same ChIP samples described in A. (D) The change in acetylation of H3K9 was determined at a subtelomere using the same ChIP samples described in (B). ChIP, chromatin immunoprecipitation; rDNA, ribosomal DNA; WT, wild type.

To determine whether Hst1 or Sir2 function at the rDNA, we conducted ChIP on acetyl histone H3. Strains lacking the deacetylases Hst1 or Sir2 are expected to have increased histone acetylation compared to wild-type cells specifically at genomic loci associated with these deacetylases. We targeted acetyl-H3K9 because *C. lusitaniae* Hst1 predominantly deacetylates this residue (Froyd *et al.* 2013). We also performed ChIP with an antibody against total H3 to control for histone levels at the examined loci. Based on the precedent in *C. lusitaniae*, Hst1 would be expected to act at the rDNA, and therefore acetylation at the rDNA would increase in *hst1*Δ*/*Δ strains but not *sir2*Δ*/*Δ strains. However, in *C. albicans*, acetylation increased in the *sir2*Δ*/*Δ strain but not the *hst1*Δ*/*Δ strain (Figure 4A). Moreover, in *C. parapsilosis*, which lacks Sir2, the loss of Hst1 did not affect acetylation of H3K9 at the rDNA (Figure 4B). These results are consistent with previous work on CaSir2 (Fu *et al.* 2008; Freire-Beneitez *et al.* 2016) and indicate that Hst1 does not act at the rDNA in either *C. albicans* or *C. parapsilosis*, in contrast to the situation in *C. lusitaniae*. Moreover, it is Sir2 that acts at the rDNA in *C. albicans*. Thus, an association with the rDNA is not a consistent feature of Hst1.

### Sir2, but not Hst1, contributes to deacetylation at telomeres

Another set of genomic locations commonly influenced by Sir2 deacetylases are the subtelomeres. In *C. albicans*, Sir2 contributes to transcriptional repression at the subtelomeres (Anderson *et al.* 2014; Freire-Beneitez *et al.* 2016). In *C. lusitaniae*, which lacks Sir2, Hst1 is not associated with subtelomeres (Froyd *et al.* 2013). These observations suggest that, after duplication, Sir2 was the paralog that retained a function at the subtelomeres. To explore this hypothesis, we determined whether Hst1 affects histone acetylation at subtelomeres in *C. albicans* or *C. parapsilosis*. As for the rDNA, we measured changes in H3K9 acetylation in strains lacking Hst1 or Sir2. In *C. albicans*, H3K9 acetylation increased in the absence of Sir2 (Figure 4C), consistent with previous studies. In contrast, no change in acetylation was observed in the absence of Hst1. Similarly, in *C. parapsilosis* no change in acetylation occurred in the absence of Hst1 (Figure 4D). Thus, in none of the three CTG species examined does Hst1 act at the subtelomeric regions.

## Discussion

A major finding of this study is that the *HST1/SIR2* gene duplicated only once in a common ancestor of the CTG clade. A single duplication is supported by two types of evidence. First, phylogenetic analysis demonstrates that Sir2 sequences are more related to one another than to Hst1 sequences (Figure 1). Second, the derived paralog, *SIR2*, is found at the same genomic location in all species examined (Figure 2). Given that *HST1* and *SIR2* arose through a single duplication, it is clear from the species tree that the *SIR2* gene was subsequently lost in at least two separate lineages (Figure 3). In addition, support for at least two independent losses was obtained through an SH-test in which all *SIR2*-containing species were constrained within a single clade. These losses in the CTG clade contrast with the situation in the *Saccharomyces* clade, in which a different duplication of *HST1*/*SIR2* led to paralogs that have been retained in all post whole-genome duplication species (Byrne and Wolfe 2005). Presumably, in the *Saccharomyces* clade both paralogs perform critical functions, whereas in the CTG clade the functions of Sir2 are dispensable or can be restored through compensatory mechanisms.

It is noteworthy that the duplication of the *HST1/SIR2* gene is quite ancient. Indeed, the clustering of CTG Hst1 proteins with *Saccharomyces* Hst1/Sir2 proteins is consistent with the duplication occurring before the CTG and *Saccharomyces* clades diverged. If so, the *Saccharomyces* clade must have lost the gene orthologous to the CTG *SIR2* gene. Thus, the retention pattern of this CTG *SIR2* gene is similar to recently described Specifically Retained Ancestral Genes (Morel *et al.* 2015) that contribute to diversity in yeast species.

A surprising observation was that neither CaHst1 nor CpHst1 acts at the rDNA array (Figure 4). In contrast, ClHst1 in *C. lusitaniae* does act at the rDNA but is not associated with any other genomic locus (Froyd *et al.* 2013; Kapoor *et al.* 2015). Thus, there is no genomic locus with which Hst1 is consistently associated in the CTG clade. Nevertheless, Hst1 is retained in all examined CTG species. One potential explanation for this retention is that Hst1 has a critical function that has yet to be discovered. This unknown function is likely to involve a nonhistone target, given that there are no common genomic targets (Kapoor *et al.* 2015).

**Figure 5:**
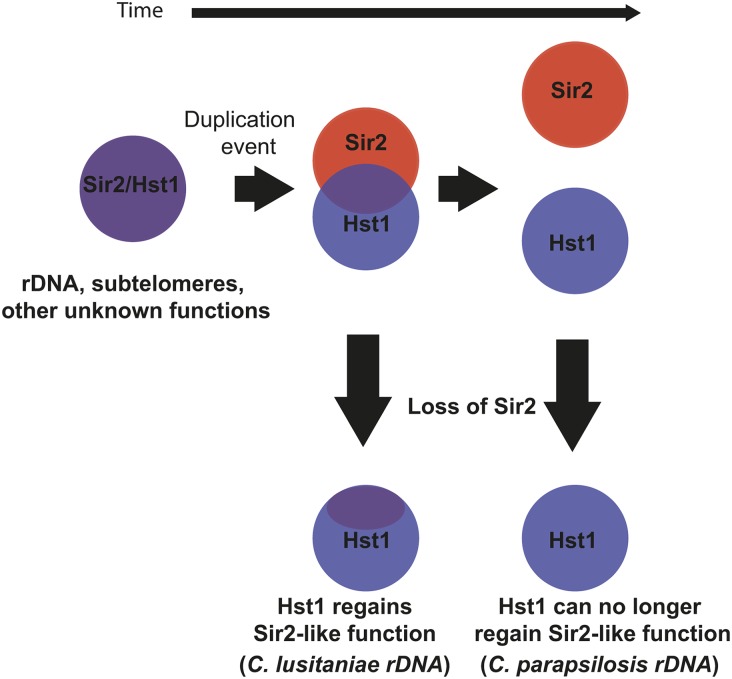
Hst1 may compensate for the loss of Sir2. After gene duplication, subfunctionalization can be a gradual process. Initially, the paralogs Sir2 and Hst1 are similar and partially substitute for one another. Loss of one paralog at this stage could provide the opportunity for the other paralog to regain the lost function. After further subfunctionalization, the paralogs may no longer substitute for one another. Loss of one paralog at this stage could not be compensated by the other paralog. rDNA, ribosomal DNA.

We also found that, in *C. albicans*, Sir2 contributes to deacetylation at the rDNA array (Figure 4). This result is consistent with CaSir2-dependent silencing of a *URA3* reporter in the rDNA (Fu *et al.* 2008; Freire-Beneitez *et al.* 2016). Nevertheless, it is surprising that the same sirtuin paralog is not associated with the rDNA in all CTG species. At least two scenarios could explain how rDNA silencing came to be mediated by Sir2 in some CTG species but by Hst1 in others. One possibility is that rapid speciation occurred following the *HST1/SIR2* duplication, allowing *HST1* and *SIR2* to diverge independently in the two lineages. In this way, separate subfunctionalization events may have led to different paralogs acting at the rDNA in the two lineages. A second scenario (Figure 5) is that, after duplication, a single subfunctionalization occurred, resulting in Sir2 acting at the rDNA. However, the subsequent loss of *SIR2* in some species created an opportunity for the paralog Hst1 to substitute for Sir2. Over time, Hst1 would have regained the ability to function at the rDNA. The feasibility of such a scenario is supported by observations that, in *S. cerevisiae*, Sir2 and Hst1 have a weak ability to substitute for one another (Xie *et al.* 1999; Hickman and Rusche 2007). Distinguishing between these possibilities will require the functional characterization of Sir2 and Hst1 proteins in additional species.

A final observation is that the loss of *SIR2* in several CTG lineages has eliminated chromatin structures that are otherwise widely conserved. For example, Sir2/Hst1 generates a distinct chromatin structure at the rDNA repeats in species as distant as *S. cerevisiae* (Gottlieb and Esposito 1989) and *S. pombe* (Shankaranarayana *et al.* 2003), as well as the CTG species *C. lusitaniae* and *C. albicans*. However, Hst1 is not present at the rDNA repeats in *C. parapsilosis* (Figure 4), and this species does not encode Sir2. In this case, perhaps Hst1 was not able to compensate for the loss of Sir2 (Figure 5). Similarly, Sir2 homologs form subtelomeric heterochromatin in a broad spectrum of species, including *S. cerevisiae* and *S. pombe*. However, Hst1 is absent at the subtelomeres of both *C. lusitaniae* (Froyd *et al.* 2013; Kapoor *et al.* 2015) and *C. parapsilosis* (Figure 4). It will be interesting to learn how these species have adapted to the loss of these sirtuin-mediated chromatin structures.

In summary, the *HST1/SIR2* genes in the CTG clade provide an interesting example in which gene duplication and diversification were followed by loss of one paralog in some species but not others. Consequently, a variety of functional outcomes are observed. In some cases, the same role can be filled by different paralogs in different species. In other cases, certain functions are lost entirely in some species. Thus, the *HST1/SIR2* genes provide fertile ground for exploring evolution of protein function.

## Supplementary Material

Supplemental Material
