# Supplementary material for: Sporadic Gene Loss After Duplication Is Associated with Functional Divergence of Sirtuin Deacetylases Among Candida Yeast Species
Source: G3 (Bethesda). 2016 Aug 18;6(10):3297–305. doi: 10.1534/g3.116.033845 (PMC5068949; doi:10.1534/g3.116.033845)
Supplement: Supplemental Material [file supp_6_10_3297__index.html]

Sporadic Gene Loss After Duplication Is Associated with Functional Divergence of Sirtuin Deacetylases Among Candida Yeast Species — Supplemental Material 

# Sporadic Gene Loss After Duplication Is Associated with Functional Divergence of Sirtuin Deacetylases Among *Candida* Yeast Species

## Supplemental Material for Rupert, *et al*, 2016

**Files in this Data Supplement:**

- Table S1 - Sir2 and Hst1 proteins used in phylogeny. (.pdf, 260 KB)
- Table S2 - Loci used in the phylogeny of CTG yeast species. (.pdf, 175 KB)
- Table S3 - Plasmids generated for this study. (.pdf, 180 KB)
- Table S4 - Primers used for qPCR analsysis. (.pdf, 181 KB)
