# Supplementary material for: Sporadic Gene Loss After Duplication Is Associated with Functional Divergence of Sirtuin Deacetylases Among Candida Yeast Species
Source: G3 (Bethesda). 2016 Aug 18;6(10):3297–305. doi: 10.1534/g3.116.033845 (PMC5068949; doi:10.1534/g3.116.033845)
Supplement: Supplemental Material [file supp_g3.116.033845_TableS1.pdf]

Table S1. Sir2 and Hst1 proteins used in phylogeny

Protein name	Accession Number
A. nidulans hst1	C8V3W5
C. albicans Hst1	XP_723447
C. albicans Hst2	KGQ81523
C. albicans Sir2	XP_719442
C. dubliniensis Hst1	XP_002417498
C. dubliniensis Sir2	XP_002418099
C. lusitaniae Hst1	XP_002617818
C. orthopsilosis Hst1	XP_003866221
C. parapsilosis Hst1	CCE41840
C. tenuis Hst1	XP_006688850
C. tropicalis Hst1	XP_002548945
C. tropicalis Sir2	XP_002546917
D. hansenii Hst1	XP_459895
G. candidum Sir2	CDO53759
K. lactis Sir2	XP_455739
K. pastoris Sir2	XP_002490032
L. elongisporus Hst1	XP_001527944
M. guilliermondii Hst1	EDK39227
M. guilliermondii Sir2	EDK40860
N. crassa Nst-1	XP_960372
R. microsporus Sir2	CEI89652
S. cerevisiae Hst1	NP_014573
S. cerevisiae Hst2	NP_015310
S. cerevisiae Sir2	NP_010242
S. coipomoensis Hst1	KX533525
S. coipomoensis Sir2	KX533530
S. passalidarum Hst1	XP_007375727
S. passalidarum Sir2	XP_007375045
S. pombe Sir2	NP_001018840
S. spartinae Hst1	KX533537
S. spartinae Sir2	KX533542
S. stipitis Hst1	XP_001387128
S. stipitis Sir2	XP_001382290
Y. lipolytica Sir2	XP_505293
