# Supplementary material for: Sporadic Gene Loss After Duplication Is Associated with Functional Divergence of Sirtuin Deacetylases Among Candida Yeast Species
Source: G3 (Bethesda). 2016 Aug 18;6(10):3297–305. doi: 10.1534/g3.116.033845 (PMC5068949; doi:10.1534/g3.116.033845)
Supplement: Supplemental Material [file supp_g3.116.033845_TableS2.pdf]

Table S2. Loci used in the phylogeny of CTG yeast species

Annotation in *S. cerevisiae*	Description
MS456 (NP_009761)	Mcm7, Mcm2-7 helicase subunit
FG533 (NP_015239)	Elp3, Elongator subunit
MS378 (NP_012056)	Kog1, Ubiquitin-binding TORC1 subunit
FG570 (NP_010066)	Gdh2, Glutamate dehydrogenase
FG595 (NP_013826)	Ilv2, Acetolactate synthase catalytic subunit
FG848 (NP_012712)	Uba1, Ubiquitin activating E1 protein
FG692 (NP_014247)	Pol1, catalytic subunit DNA polymerase alpha
FG610 (NP_012526)	Cct8, Chaperonin Cct ring complex subunit
MS407(NP_010476)	Rvb1, RUVB family ATP-dependent DNA helicase
MS431(NP_010016)	Msh3, Mismatch repair protein
