# Supplementary material for: Sporadic Gene Loss After Duplication Is Associated with Functional Divergence of Sirtuin Deacetylases Among Candida Yeast Species
Source: G3 (Bethesda). 2016 Aug 18;6(10):3297–305. doi: 10.1534/g3.116.033845 (PMC5068949; doi:10.1534/g3.116.033845)
Supplement: Supplemental Material [file supp_g3.116.033845_TableS3.pdf]

Table S3. Plasmids generated for this study

Plasmid	Oligonucleotides used	Restriction sites
C. albicans HST1 (pLR0984) *HST1* from SN152 in pRS316	CCCAAGCTTCCTCACCCTTTCCTCTATTC	HindIII, XbaI
C. albicans hst1Δ::ARG4 (pLR0998) A*RG4* from pSN69 in pLR0984	CCACCACCACTTCTACCACCGGGAATCTCGGTCGTAATG<br>CTCTTTGTCCTCGTTGTCCACGACTCACTATAGGGAGACCGGC	
C. albicans SIR2 (pLR0983) *SIR2* from SN152 in pRS316	CCATCGATGGTGGTATCTTGGATTCAACC	SpeI, ClaI
C. albicans sir2Δ::ARG4 (pLR0996) *ARG4* from pSN69 in pLR0983	GAGTGAGTGAGTGGAGTAGCGCGGGAATCTCGGTCGTAATG<br>CACAAAGATACCCAAACTCCTATCGACTCACTATAGGGAGACCGGC	
C. parapsilosis HST1 (pLR1070) *HST1* from CPL2H1 in pRS416	GCGCCGCGGCTATGCGGATTGAGAGCGTC	HindIII, SacII
C. parapsilosis hst1Δ::HIS1 (pLR1136) *HIS1* from pSN52 in pLR1070	GCAGGAATCTGCATCACAATAGCGCCAAATACTATTAACCTAAGTCCGCCAGTGTGCTGG<br>TTCACTCGCAACCTCGAACATTAACACGATCATCTCTTCCAGTGTGATGGATATCTGCAG	
C. parapsilosis hst1Δ::LEU2 (pLR1137) *LEU2* from pSN40 in pLR1070	GCAGGAATCTGCATCACAATAGCGCCAAATACTATTAACCTAAGTCCGCCAGTGTGCTGG<br>TTCACTCGCAACCTCGAACATTAACACGATCATCTCTTCCAGTGTGATGGATATCTGCAG	
