# Supplementary material for: Sporadic Gene Loss After Duplication Is Associated with Functional Divergence of Sirtuin Deacetylases Among Candida Yeast Species
Source: G3 (Bethesda). 2016 Aug 18;6(10):3297–305. doi: 10.1534/g3.116.033845 (PMC5068949; doi:10.1534/g3.116.033845)
Supplement: Supplemental Material [file supp_g3.116.033845_TableS4.pdf]

Table S4. Primers used for qPCR analsysis

Species	Locus	Sequence
C. albicans	rDNA 1	TGGCAGTCAAGCGTTCATAG
	rDNA 1	CAGCCGCAAAAACCAATTAT
	rDNA 2	GCACTCGGTCACTTGACAC
	rDNA 2	GGCCGAAGTTAACCTGTTGC
	rDNA 3	GGGGTTGGGTGGAAATAAGG
	rDNA 3	CAACCACCAACCACCAACC
	rDNA 4	CTGGGGATAGACGTTTATGG
	rDNA 4	GTGACTAGCATCAGCTAGTG
	rDNA 5	GTGGTCTGTTTTAAGTTGTTTTGTGTAG
	rDNA 5	CAATTAGATGGCAGTGTTTTCGTTTAAAG
	rDNA 6	GAGAATACTAGTAATTTTTTGTATGTGTGCAC
	rDNA 6	CCATACAAAAAATTACTATGTATTCTCACACAC
	rDNA 7	GGGTCGATTACCCTTGGTTC
	rDNA 7	GTTCCCCGTTCGATCAACC
	rDNA 8	CACCAGTGGTGTTACACAGCC
	rDNA 8	CCAGGCATAATTGTGGTTGCC
	rDNA 9	GTGGGTCACGTGCATTTAATTAGG
	rDNA 9	GTAGCGATGAGGACGTGTAG
	orf19.2926 / orf19.2927	GTGGTGGCCGTTTATAAGTGTG
	orf19.2926 / orf19.2927	GACCCCCACCTTACTACTAC
	Subtelomere 3L	GAGCCACACAGATTCGACAG
	Subtelomere 3L	CAATCACGGATGGCTAGACAG
	Subtelomere 3R	GGAGAAGAAGCTGGAGAGTAC
	Subtelomere 3R	GTGTCCCAGTCACACTCAAG
C. parapsilosis	rDNA 1	CTGTTTCCGGTGGTGTCG
	rDNA 1	GCCATGTTAAACCCTGGG
	rDNA 2	CGTGGCCTTAAACAACTGG
	rDNA 2	GCGAATTTTCTCCTGGGG
	rDNA 3	GGAGGTAGGTTGTCGTGACG
	rDNA 3	GATATGTGCGACCACTCTCC
	rDNA 4	CCATACGCGAAACTCTCG
	rDNA 4	CGACTCTGAGCATCCACC
	rDNA 5	GGCTTACCCCCCTGTTG
	rDNA 5	CGTTGCCAGGGTTGAGTG
	PRI2	CTTGCCCTTGCTCAACC
	PRI2	CCCAATTGTTGACGTCCC
	Subtelomere 006372L 1	GTGGCACTCAAACCAACC

Subtelomere 006372L 1	CAATACCCGTGCTGCCTC
Subtelomere 006372L 2	CCGCGACAACGACTTGTC
Subtelomere 006372L 2	CCACTACACCAATCGTCC
